# Collagen 1a1 Expression by Airway Macrophages Increases In Fibrotic ILDs and Is Associated With FVC Decline and Increased Mortality

**DOI:** 10.3389/fimmu.2021.645548

**Published:** 2021-11-17

**Authors:** Eliza Tsitoura, Athina Trachalaki, Eirini Vasarmidi, Semeli Mastrodemou, George A. Margaritopoulos, Maria Kokosi, Dionysios Fanidis, Apostolos Galaris, Vassilis Aidinis, Elizabeth Renzoni, Nikos Tzanakis, Athol U. Wells, Katerina M. Antoniou

**Affiliations:** ^1^ Laboratory of Molecular and Cellular Pneumonology, Department of Respiratory Medicine, School of Medicine, University of Crete, Heraklion, Greece; ^2^ Interstitial Lung Disease Unit, Royal Brompton and Harefield Hospital National Health Service (NHS) Foundation Trust, Imperial College, London, United Kingdom; ^3^ Division of Immunology, Alexander Fleming Biomedical Sciences Research Centre, Athens, Greece

**Keywords:** IPF, RA-ILD, NSIP, airway macrophages, SPP1, osteopontin, collagen 1A1, PF-ILD

## Abstract

Within the Interstitial Lung Diseases (ILD), patients with idiopathic pulmonary fibrosis (IPF) and a subset of those with non-IPF fibrotic ILD have a distinct clinical phenotype of progression despite management. This group of patients has been collectively termed the progressive fibrotic phenotype (PFP). Their early recognition may facilitate access to antifibrotic therapies to prevent or slow progression. Macrophages/monocytes within the lung orchestrate the progression and maintenance of fibrosis. A novel role for monocyte-derived macrophages during tissue damage and wound healing is the expression of collagens. We examined Collagen 1a1 expression in airway macrophages from ILD patients at diagnosis. COL1A1 mRNA levels from BAL cells were elevated in IPF and Non-IPF patients. The presence of a UIP pattern and a subsequent progressive phenotype were significantly associated with the higher BAL COL1A1 levels. In Non-IPF patients, higher COL1A1 levels were associated with a more than twofold increase in mortality. The intracellular localisation of COL1A1 in airway macrophages was demonstrated by confocal microscopy in CD45 and CD163 co-staining assays. Additionally, airway macrophages co-expressed COL1A1 with the profibrotic SPP1 gene product osteopontin. The levels of SPP1 mRNA and OPN in the BAL were significantly higher in IPF and Non-IPF patients relative to healthy. Our results suggest that profibrotic airway macrophages are increased in the BAL of patients with IPF and other ILDs and co-express COL1A1 and OPN. Importantly, COL1A1 expression by pro-fibrotic airway macrophages could be a marker of disease progression and poor survival in ILDs.

## Introduction

Interstitial Lung Disease (ILD) is a broad term currently used to include more than 200 different disease entities (1). Connective tissue associated ILD (CTD-ILD) is the most common subtype, whilst idiopathic ILDs lie within the orphan characterization of rare lung diseases. Amongst idiopathic interstitial pneumonias, the most characterized and well-studied is IPF, a lethal chronic disease (1). IPF is distinguished by a clinical phenotype of inexorable progression and a median survival of 3 years, prior to the advent of anti-fibrotic agents (2). Amongst other ILDs, disease behaviour is strikingly diverse, ranging from self-limited, reversible to progressive, IPF-like disease (1–3). A subset of non-IPF ILD patients exhibit a more progressive disease course despite usual management, similar to IPF (PF-ILD) (3–5).

Commonalities in clinical behaviour in IPF and non-IPF ILDs with the PFP suggest common underlying pathogenetic pathways that drive progression, irrespective of the initial trigger (6, 7). Given the strong links between progression and subsequent mortality in non-IPF ILDs, it is vital that algorithms be developed to identify the PFP at presentation. It is known that a pattern of usual interstitial pneumonia (UIP), on biopsy or as judged by CT (UIP or “probable UIP”) indicates a higher likelihood of disease progression (8, 9). Molecular biomarkers have also been identified as candidate markers of progression in individual ILDs, including KL-6, PDGF, FGF, VEGF and M-CSF (7, 10).

The role of the innate and adaptive immune systems in the pathogenesis of IPF is not clearly defined (11) and immune cells such as macrophages, monocytes and fibrocytes are subject to intense investigation as novel therapeutic options in IPF effectively alter their activation (12, 13). Macrophages, the most abundant immune cells in the lung, play important roles in tissue remodelling during pulmonary fibrosis (14). Following a fibrotic insult such as bleomycin in murine models, the pool of tissue resident alveolar macrophages is replaced by monocyte-derived macrophages that have distinct phenotypes including the upregulation of pro-fibrotic genes (15). Macrophages produce profibrotic mediators such as transforming growth factor-β (TGF-β) and platelet-derived growth factor (PDGF), directly orchestrating the activation of fibroblast functions and implicated in the aberrant wound-healing cascade during fibrosis (16). Fibrocytes, a minor fraction of circulating leukocytes, portray a dual phenotype of monocytes (CD34, CD45, CD11b) and fibroblasts (collagens I and III, and fibronectin (17) that migrate to the lungs following tissue damage (18). Single-cell RNAseq analyses of the airway cell population from human lungs reveal a plethora of cell entities with monocyte and macrophage markers. This approach is reshaping the field of macrophage/monocyte biology, with the observation that lung macrophage/monocyte populations shift during lung fibrosis towards distinct entities with yet uncharacterised functions (19).

Macrophages were long believed not to produce collagens. However there is cumulative evidence that they express almost all known collagen mRNAs (20, 21). Furthermore, recent evidence from a mouse model of fibrotic scar formation in the heart shows that recruited monocyte-derived macrophages directly contribute to collagen-I fibril formation, suggesting that cell-autonomous production of collagen is a component of the pro-fibrotic monocyte/macrophage response (22). The role of collagen expression by alveolar macrophages in IPF is unknown; however, a recent study showed that Collagen VI is upregulated in macrophages overexpressing Fra-2 in mouse models of pulmonary fibrosis and in IPF tissue macrophages while Col-VI knockout bone marrow chimeras were protected from bleomycin-induced lung fibrosis (23). We have previously observed that mRNA levels of COL1A1 are detectable in BAL cells and are elevated in IPF patients relative to controls (24, 25).

The results of the lung fibrosis cell atlas suggest that macrophage populations in the lungs are distinct in lung fibrosis compared to healthy subjects. Independent studies have identified an increase of alveolar macrophages expressing the *SPP1* gene that encodes for osteopontin (OPN) in fibrotic lungs (15, 19, 26). OPN-expressing macrophages of monocyte origin also accumulate in murine models of hepatosteatosis (27). OPN dramatically increased in the BAL and serum of fibrotic patients and was initially explored as a potential IPF serum biomarker (28, 29). However, results from later studies established a wider pattern of upregulation of OPN in fibrotic disease suggesting a dominant role in the pathogenesis of lung fibrosis (30). OPN may act as a cytokine and a transcription factor and is directly involved in extracellular matrix regulation and collagen expression in fibrotic disorders of the liver, kidneys and heart (31, 32).

In this study, we established that collagen1a1 expression occurs in airway macrophages and increases in pulmonary fibrosis both in mice and humans. We compared the COL1A1 mRNA levels between IPF and non-IPF fibrotic ILDs at diagnosis in order to establish a possible link between disease progression and collagen expression in myeloid cells. In addition, in view of the involvement of OPN in collagen expression, we also examined a possible association of SPP1 and COL1A1 levels in BAL cells.

## Materials and Methods

### Patients

134 patients were prospectively recruited. 69 subjects (ILD, n=50; controls, n=19) were recruited in the Department of Respiratory Medicine, University Hospital of Heraklion, Crete, Greece from May 2012 to December 2018. 65 subjects all with ILD, were recruited in the Royal Brompton Hospital from March 2003 to October 2009.

Patient characteristics are summarised in **Table 1** and **Supplementary Table 1**. Diagnostic sub-groups comprised IPF (n=53) and non-IPF ILDs (n=52). All subjects underwent bronchoalveolar lavage (BAL) and chest high resolution computed tomography (HRCT). For the ILD patients BAL and HRCT were part of the initial diagnostic evaluation (33–36).

**Table 1:** Patient characteristics.

	Control Group (n = 19)	IPF (n = 53)	Non-IPF (n = 62)	P value
Age	54.1 ± 13.5	68.1 ± 10	59 ± 14	P<0.0001 Control *vs* IPF p=0.001 IPF *vs* Non-IPF p<0.001 Control *vs* Non-IPF ns
gender (female/male)	6/13	8/45	41/21	P<0.0001 *
Smoking History				P<0.0001*
Never	2 (10.5%)	16 (30.8%)	35 (57.4%)	
Ex-smoker	3 (15.8%)	31(59.6%)	6 (32.8%)	
Smoker	14 (73.7%)	5 (9.6%)	20 (9.8%)	
Macrophages	87.3 ± 9	79.1 ± 15.3	79 ± 12.6	P ns
Lymphocytes	9.9 ± 7.6	11 ± 15.2	11 ± 9	P ns
Neutrophils	4.2 ± 1.6	7.3 ± 6.3	6.5 ± 6.2	P=0.006 Control *vs* IPF p<0.001 Control *vs* Non-IPF p<0.001
Eosinophils	0.4 ± 0.5	2 ± 3.3	2.3 ± 2.8	P=0.087 Control *vs* IPF p=0.007 Control *vs* Non-IPF p<0.0001
FVC		78.5 ± 19.7	79.4 ± 25.2	P ns
DLco		52.3 ± 18.5	51 ± 16.5	P ns
CPI		44 ± 15	43 ± 14.7	P ns

*Stands for Chi-square test; “ns” stands for non significant.

#### IPF Group

The diagnosis of IPF was based on ATS/ERS criteria or on multidisciplinary discussion according to the Fleischer criteria (34, 37). Patients were anti-fibrotic naïve.

#### Non-IPF Group

This category of patients included patients with a known or new diagnosis of Connective tissue disease (CTD-ILD); namely Systemic Sclerosis (SSc), Rheumatoid arthritis (RA), Systemic Lupus erythematous (SLE) or Undifferentiated CTD. Patients with CTD-ILD were enrolled at the initial stages of there ILD diagnosis. Additionally, included patients with Idiopathic Non-Specific Pneumonia (NSIP), Hypersensitivity Pneumonitis (HP) and organizing pneumonia/NSIP overlap (OP/NSIP).

#### Control Group

Control subjects were undergoing bronchoscopy for the investigation of haemoptysis, without any overt pulmonary comorbidities and with normal bronchoscopy findings and cytology results. Since controls were healthy subjects, no PFTs were performed.

All patients were evaluated with complete pulmonary function tests (PFTs) within one month of bronchoscopy. Lung volumes were measured using body plethysmography and the diffusion capacity (DLco, corrected for haemoglobin) was measured using the single breath technique. The computerized system (Jaeger 2.12; MasterLab, Würzburg, Germany) was used and predicted values were obtained from the standardized lung function testing of the European Coal and Steel Community, Luxembourg (1993).

Patients were classified as non-smokers, current smokers or former smokers (defined as having smoked a minimum of one cigarette a day for a minimum of 1 year, stopping at least 6 months before presentation).

All patients provided written informed consent. The study was approved by the Ethics Committees of the University Hospital of Heraklion (IRB number: 1045 and 17030) and the Royal Brompton Hospital (REC reference 13/LO/0857).

### BAL Cell Isolation and Determination of Cellular Composition

BAL fluid was obtained at room temperature. A flexible bronchoscope was wedged into a sub-segmental bronchus of a predetermined region of interest based on radiographical findings. A BAL technique was performed by instilling 180 ml of normal saline in 60-mL aliquots, retrieved by low suction. BAL samples were kept on ice and processed within two hours of collection. Samples were filtered through sterile 70nm cell strainers (BD) and centrifuged at 500g for 5 minutes at 4°C. Cell pellets were re-suspended with cold PBS. Total cell count and cell viability were assessed using Trypan blue (ICN). Differential cell population count was analysed following May-Grunewald-Giemsa staining (25).

### Mice

Mice were bred under SPF conditions at the local animal facility at “20–22°C, 55 ± 5% humidity, and a 12-h light/dark cycle; water and food were given ad libitum”. All experimentation in mice, in line with the ARRIVE guidelines, was approved by the Veterinary service and Fishery Department of the local governmental prefecture (#2816), following the positive opinion of the Institutional Protocol Evaluation Committee of BSRC Alexander Fleming.

Pulmonary fibrosis was induced through the administration of 0.8U/Kg of bleomycin (Nippon Kayaku) to anesthetized mice (IP ketamine/xylazine/atropine, 100/10/0.05 mg/kg, respectively) *via* the oropharyngeal (OA) route and bronchoalveolar Lavage Fluid (BALF) was collected as previously described (38). In brief, anaesthetized mice were stabilized on a plastic wall. The tongue was carefully pulled out in order to get a clear view of the trachea and at the same time, the nares were blocked to force bleomycin inhalation. The appropriate volume of bleomycin diluted in normal saline (~50μL for each mouse) was directly delivered in the oropharyngeal cavity using a conventional pipette tip. At day 14 after bleomycin administration mice were euthanized and bronchoalveolar fluid was obtained by lavaging the airways with 3mL of normal saline using a cannula through the trachea (three times, 1mL each). Then, BALF cells were collected by a 15 min centrifugation at 1.200rpm/4°C. Cell pellets were resuspended in NucleoZol (Macherey-Nagel, 740404.200) for isolation of total RNA as specified by the manufacturer. SuperScript ™ IV VILO™ (Invitrogen, 11766050) was used following the manufacturers guidelines for reverse transcription.

Quantitative real-time polymerase chain reaction (QRT-PCR) was performed using SoFAst EvaGreen Supermix on a Bio-Rad CFX96 Touch™ Real-Time PCR Detection System (Bio-Rad Laboratories Ltd, CA, USA).Values were normalized to β2-microglobulin (B2M).

### RNA Extraction and mRNA Expression

1-1.5 million cells were centrifuged and cell pellets were homogenised in TriReagentTM(MBL) for total RNA, followed by storage at -80°C. Total RNA was isolated as previously described (25). Oligos for RT-PCR amplifications were retrieved from http://www.universalprobelibrary.com. COL1A1: Fwd:5’GGGATTCCCTGGACCTAAAG 3’, Rev: 5’ GGAACACCTCGCTCTCCA 3’, SPP1: Fwd: 5’ GGGCTTGGTTGTCAGCAG 3’, Rev: 5’TGCAATTCTCATGGTAGTGAGTTT 3’, GAPDH: Fwd: 5’AGCCACATCGCTCAGACAC3’, Rev: 5’GCCCAATACGACCAAATCC 3’. Primer pairs correspond to gene specific assays #67, 63, and 60 respectively. GAPDH levels were used as an endogenous control for the normalization of mRNA expression levels in BAL samples. Gene expression analysis was performed following incorporation of relative expression values in average (duplicates) normalized by GAPDH. Relative expression values for the patient cohort were calculated by 2-^ΔΔCt^ method, where ΔΔCt =(sample Ct GOI-sample Ct GAPDH)-(Calibrator Ct GOI- Calibrator Ct GAPDH), GOI= gene of interest, calibrator= mean of all Cts. Relative gene expressions were log transformed.

Mouse Q-Real-time PCR was performed on a BioRad CFX96 TouchReal-Time PCR Detection System (Bio-Rad Laboratories Ltd, CA, USA). For the detection of collagen 1α1 transcript we used the following primers: 5’-CTACTACCGGGCCGATGATG-3’ (F) & 5’-CGATCCAGTACTCTCCGCTC-3’ (R). Values were normalized to the expression of b-2 microglobulin [B2M, primers: 5’-TTCTGGTGCTTGTCTCACTGA-3’ (F) & 5’-CAGTATGTTCGGCTTCCCATTC-3’ (R)].

### Airway Macrophage Cell Culture

0.5 million freshly isolated BAL cells were cultured in DMEM, high glucose, with stable glutamine and sodium pyruvate (Biosera) supplemented with 2% Fetal Calf Serum (FCS) (Biosera) and 1x concentration of penicillin-streptomycin (from 100x concentrated solution, Biosera) in a humidified incubator at 37°C containing 5% CO_2_ for 30min, with washes to remove non-adherent cells, such as lymphocytes and eosinophils. In order to avoid changes in gene expression caused by the contact of cells with rigid surfaces that may lead to detectable altered protein levels of COL1A1 or OPN, cells were cultured for a short period of thirty minutes. Cells were fixed with 4% formaldehyde (FA) for 20 min at RT and were stored overnight in 1% FA.

### Immunofluorescence

FA fixed cells were washed three time with PBS and permeabilization buffer (0.5%FBS, 0.2%Triton in TBS) was added for 10 minutes followed by antibody blocking buffer (0.5%FCS, 0.1%Triton, 2mg/ml BSA in TBS) for 10 minutes at RT. Primary antibody incubations were carried out for 60 minutes at room temperature in antibody blocking buffer followed by washing with TBS. Secondary antibody incubations were performed for 30 minutes followed by washing with TBS. ToPro-633 or DAPI was added for nuclear staining. Finally, TrueBlack (Biotium) was added for 30 seconds to eliminate autofluorescence signal. COL1A1 was detected with rabbit anti-Col1a1 antibody (TA506380, Boster biologicals) at final concentration 5μg/ml, CD45 with mouse anti-CD45 (M0701, Dako) diluted 1/100 times, CD163 with mouse anti-CD163 (TA506380, Origene) at final concentration 3.5μg/ml, osteopontin was detected with mouse anti-OPN (MAB1433, RnD systems) at final concentration 3μg/ml. Secondary antibodies were: for CD45 and CD163 staining, anti-Mouse Cy3 (M 30010, Thermo Scientific), for OPN staining of fresh BAL cultures Alexa-Anti-mouse 633 (A-21126 Thermo Scientific) or Alexa anti-mouse 488(A11001, Thermo Scientific) and for COL1A1, Alexa-anti-rabbit 488 (A11008, Thermo Scientific). All secondary antibodies were used in 1/500 dilution. Secondary antibody controls were also performed with no background fluorescence. Images were taken by Sp2 Leica confocal microscopy with 63x Plan-Apochromat oil lens. Mean intensity of protein levels/cell were calculated with ImageJ v2.0.0-rc-69/1.52i as follows, photomicrographs, with identical acquisition settings, were adjusted for background, AMs were contoured and mean pixel signal intensity was measured for each cell. 5-8 photomicrographs, depending on cell density and an average of 100 cells per patient sample were measured.

### Statistical Analysis

Data were analysed using Prism 8 (Graph Pad) software. Gene expression comparisons between two groups of a normal distribution were performed with Welch’s T test, when sample numbers between the populations were unequal or a t-test when population numbers were similar When populations were of a not normal distributions Mann-Whitney test was performed. Gene expression comparisons between more than two groups were performed with one-way Anova and multiple comparisons were performed with Two-stage linear step-up procedure of Benjamini, Krieger and Yekutieli recommended in Prism 8 software. Comparisons of intracellular protein levels/cell obtained by immunofluorescence between the groups were performed with Kruskal-Wallis test and multiple comparisons were performed with Two-stage linear step-up procedure of Benjamini, Krieger and Yekutieli recommended in Prism 8 software.

Survival was analysed using SPSS 25 (IBM) software. Survival analysis was performed using Cox proportional hazard analysis. We used receiver operating characteristics (ROC) curve analysis to select a cut point for COL1A1 that predicted progression and generated hazard ratios (HRs) to compare patients assigned to groups based on the optimal cut-point. A *p* value less than 0.05 was considered statistically significant (*p<0.05, **p<0.01, ***p<0.001, ****p<0.0001).

## Results

### COL1A1 mRNA Expression Was Elevated in the BAL Cells From IPF Patients and Experimental Fibrosis in Mice

**Figure 1:**
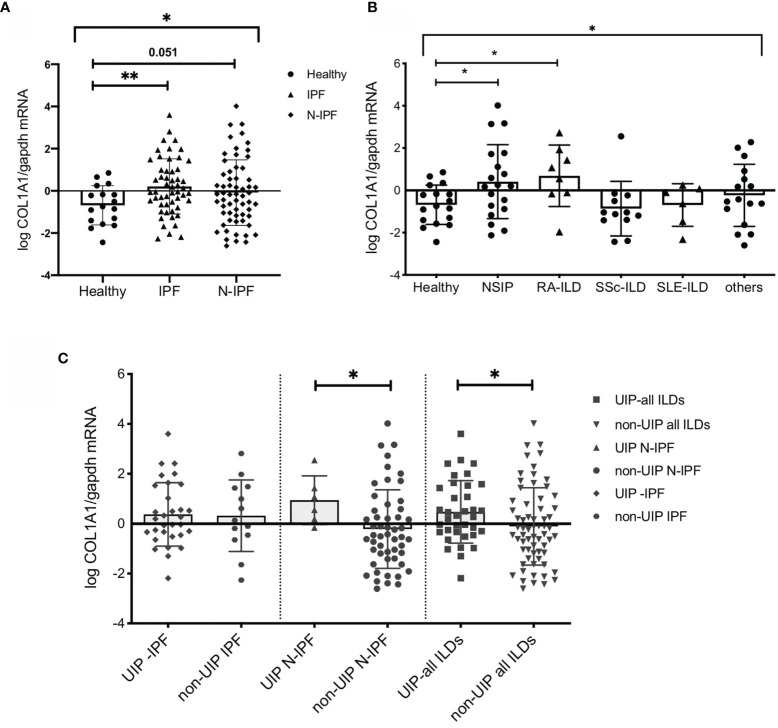
COL1A1 mRNA expression is upregulated in BAL cells from fibrotic ILDs and is higher in ILD patients with UIP *versus* non-UIP patterns of interstitial pneumonia. COL1A1 relative mRNA levels in: **(A)** IPF and N-IPF relative to healthy subjects, (One way Anova test and pairwise t tests), **(B)** COL1A1 expression in whole BAL cells in the ILD diagnostic subgroups relative to heathy. One-way Anova, and pairwise Welch’s T tests, p = 0.024), and H *vs* RA p = 0.031 (or Welch’s T test p = 0.035). **(C)** Definitive UIP *versus* all other non-UIP patterns in IPF, N-IPF and all ILD patients included in the study. (*p < 0.05, **p < 0.01).

We have previously observed that mRNA levels of COL1A1 are detectable in BAL cells and are elevated in IPF patients relative to controls (24, 25). Whole BAL cell analyses by RT-PCR in the current cohort confirmed the significant upregulation of COL1A1 mRNA in IPF compared to controls (p=0.0036) (**Figure 1** **A**). Similar results could be obtained by an alternative approach, using microarray transcriptomic data from BAL cell samples of IPF patients (39). Re-analysis of the above dataset showed that COL1A1 was among the significantly upregulated genes that increased more than 2 fold relative to healthy individuals (0.41 log2FC; 0.0007 p value; 0.008 FDR corrected p value) (**Supplementary Figure 1A**).

We also measured COL1A1 expression by RT-PCR in BAL cells from bleomycin treated mice and found that COL1A1 mRNA was induced by the bleomycin treatment (**Supplementary Figure 1B**). In agreement with our results, re-analysis of RNAseq data from alveolar macrophages isolated from mice fourteen days following bleomycin instillation (40), showed significant upregulation of co1a1 relative to saline treated mice (3.41 log2FC; 1.38e-06 value; 4.4e-05 FDR corrected p value) (**Supplementary Figure 1C**).

### COL1A1 mRNA Expression Was Elevated in the BAL Cells From Non-IPF ILD Patients and in Particular Those With UIP Pattern on CT

COL1A1 expression in the BAL was upregulated during fibrosis in both humans and animal models. We therefore sought to examine whether COL1A1 upregulation was specific to IPF or was a characteristic of Non-IPF ILD patients as well. Whole BAL cell mRNA analysis showed that COL1A1 mRNA levels were higher not only in IPF but showed a similar trend in other ILD diagnostic subgroups, collectively compared to controls (one-way Anova test: p=0.03 and Welch’s t-test pairwise comparisons, IPF relative to healthy: p=0.036, N-IPF relative to healthy: p=0.051) (**Figure 1** **A**). Within the non-IPF ILD subgroup, significant differences were observed between the groups, (One-way Anova test: p= 0.04) and in particular RA-ILD and NSIP, showed significantly increased COL1A1 mRNA expression relative to healthy individuals following pairwise comparisons (t-test, p=0.035 and p=0.024 respectively) (**Figure 1** **B**).

Next, we examined the expression of COL1A1 according to fibrosis pattern on CT, and found that in the whole ILD cohort a UIP pattern was associated with higher COL1A1 levels compared to all other patterns combined (Welch’s t-test, p=0.04) (**Figure 1** **B**). Importantly, within the Non-IPF cohort, patients with a UIP pattern of fibrosis had significantly higher levels of COL1A1 expression (t-test, p=0.04) whilst, in the IPF group, no significant difference was observed between patients with UIP when compared to probable UIP (**Figure 1** **C**).

### Airway Macrophages Express Intracellular COL1A1 in Fibrotic ILDs

**Figure 2:**
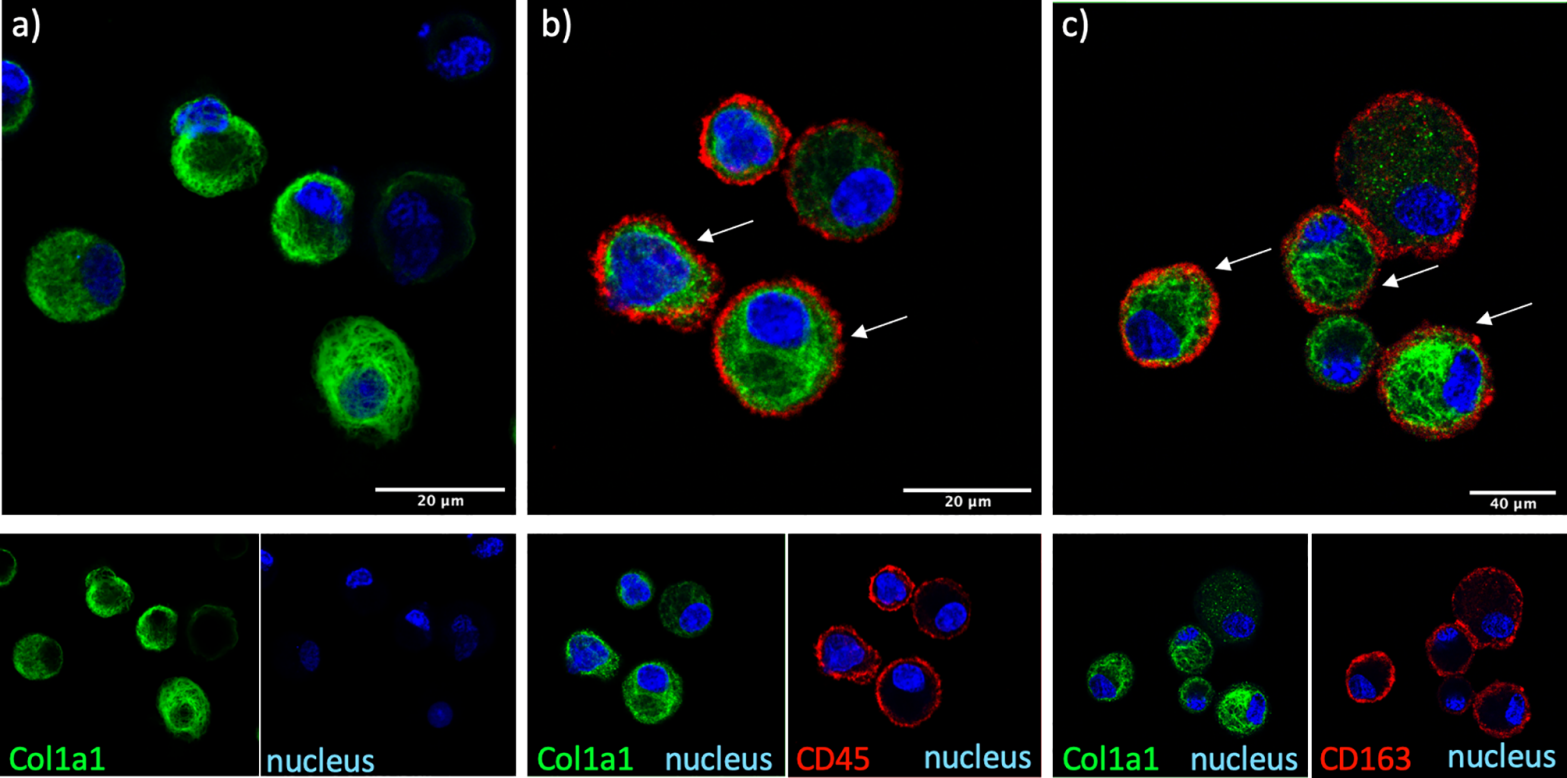
COL1A1 is expressed by cells of myeloid origin and AMs. COL1A1 expression in fresh BAL cultures from a Non-IPF patient with CTD-ILD, stained with rabbit anti-human-COL1A1 and ToPro-633 nuclear stain and **(A)** mouse isotype control, **(B)** mouse anti-CD45 and **(C)** mouse anti-CD163 antibodies. Arrows indicate CD45 and CD163 positive cells expressing COL1A1.

The cellular source of COL1A1 in the BAL was examined by immunofluorescence in freshly isolated AMs from BAL of IPF and non IPF f-ILD patients. COL1A1 staining demonstrated a characteristic ER localization, illustrated by a membranous web in the cells (**Figure 2**). Some extent of phagocytosis could not be excluded as COL1A1 positive cytoplasmic vacuoles were also detectable. Furthermore, to confirm that COL1A1 positive cells were of myeloid origin and to exclude the possibility that undetected fibroblasts were responsible for the COL1A1 mRNA measured, cells were co-stained with anti-CD45 a pan-leukocyte marker. As shown in **Figure 2B**, CD45 positive cells showed high COL1A1 expression confirming the expression of COL1A1 by leukocytes. Airway macrophage identity of COL1A1 stained cells was subsequently demonstrated by the co expression of macrophage scavenger receptor CD163 on their surface (**Figure 2** **C**), which we have previously shown to be elevated in IPF AMs (24).

### Intracellular COL1A1 Is Elevated in AMs From f-ILDs

**Figure 3:**
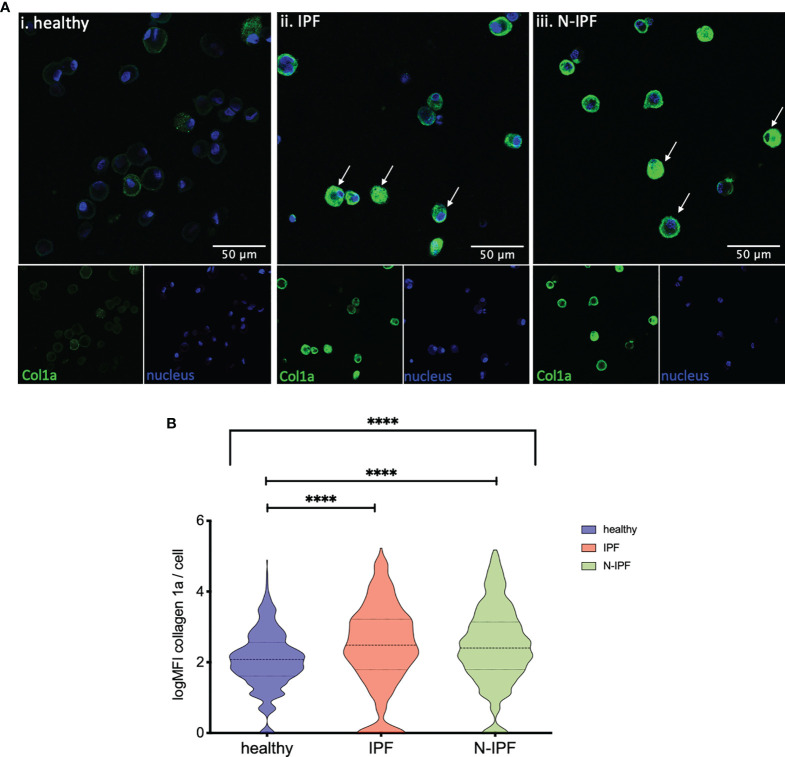
Increased expression of COL1A1 in AMs from fibrotic ILDs. **(A)** Typical images of BAL cytospins stained with anti-human COL1A1 and ToPro-633 nuclear stain from (i) healthy, (ii) IPF, and (iii) a representative N-IPF (RA-ILD) sample. Arrows indicate prominent COL1A1 expression in BAL cells. **(B)** Violin plots of mean log COL1A1 expression/cell per group. (Brown-Forsythe Anova, with pairwise comparisons, ****p < 0.0001).

Furthermore, COL1A1 protein levels were semi-quantified by immunofluorescence in formalin fixed whole BAL cell cytospins from samples of IPF, N-IPF and healthy subjects. As shown in **Figure 3** **A**, COL1A1 expression was heterogeneous among the BAL cells, and was strongly elevated in cells displaying airway macrophage morphology in f-ILD patients. The mean expression of COL1A1/cell calculated from all samples tested per group showed that IPF and N-IPF derived BAL cells had significantly higher COL1A1 expression than healthy subjects (**Figure 3** **B**). The mean expression of COL1A1/cell for each patient tested, as well as within non-IPF diagnostic subgroups is shown in **Supplementary Figure 2**.

### SPP1/OPN Is Elevated in BAL Cells From F-ILDs and Co-Expressed With COL1A1 in AMs

**Figure 4:**
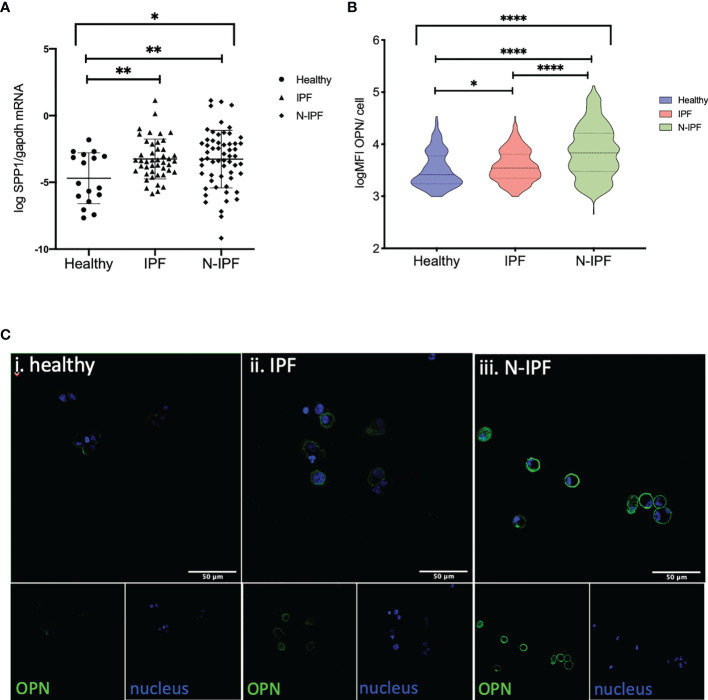
SPP1 and OPN expression in BAL cells. **(A)** SSP1 relative mRNA levels in IPF and N-IPF relative to healthy (Brown-Forsythe Anova, with individual comparisons), **(B)** Violin plots of mean OPN expression/cell per disease group (Kruskal-Wallis test and individual comparisons) and **(C)** Typical Images of BAL cytospins stained with anti-human OPN antibody and ToPro-633 nuclear stain in (i) healthy, (ii) IPF and (iii) N-IPF samples (*p < 0.05, **P < 0.01, ****p < 0.0001).

The *SPP1* gene encodes for the cytokine OPN that regulates COL1A1 expression and independent recent studies have shown an increase in a population of pulmonary macrophages expressing SPP1 mRNA in IPF and other ILDs (26). As such, we tested if COL1A1 expression was associated with SPP1 expression in BAL cells. Our results confirmed the increase of SPP1 mRNA in whole BAL cell extracts in IPF relative to healthy subjects however, we found that SPP1 was also significantly elevated in N-IPF relative to both healthy and IPF (Kruskal-Wallis test: p<0.0001, pairwise comparisons IPF relative to healthy: p=0.02, N-IPF relative to healthy: p<0.0001 and N-IPF relative to IPF p: <0.0001) (**Figure 4A**). Within the fibrotic ILDs diagnostic subgroups, NSIP, RA-ILD and SLE-ILD BAL cells had significantly higher SPP1 levels relative to healthy (**Supplementary Figure 3**).

The expression of OPN was evaluated by immunofluorescence in whole BAL cytospins. AMs in N-IPF patients showed particularly high levels of OPN expression when compared to controls (**Figure 4** **B**), as were NSIP and RA-ILD (**Supplementary Figure 4**). OPN was detected mainly in the outer membrane of AMs, although high levels of cytoplasmic expression were also observed in a fraction of cells in the fibrotic patients (**Figure 4** **C**). Neutrophils also stained positive for OPN in f-ILDs (**Supplementary Figure 5**).

**Figure 5:**
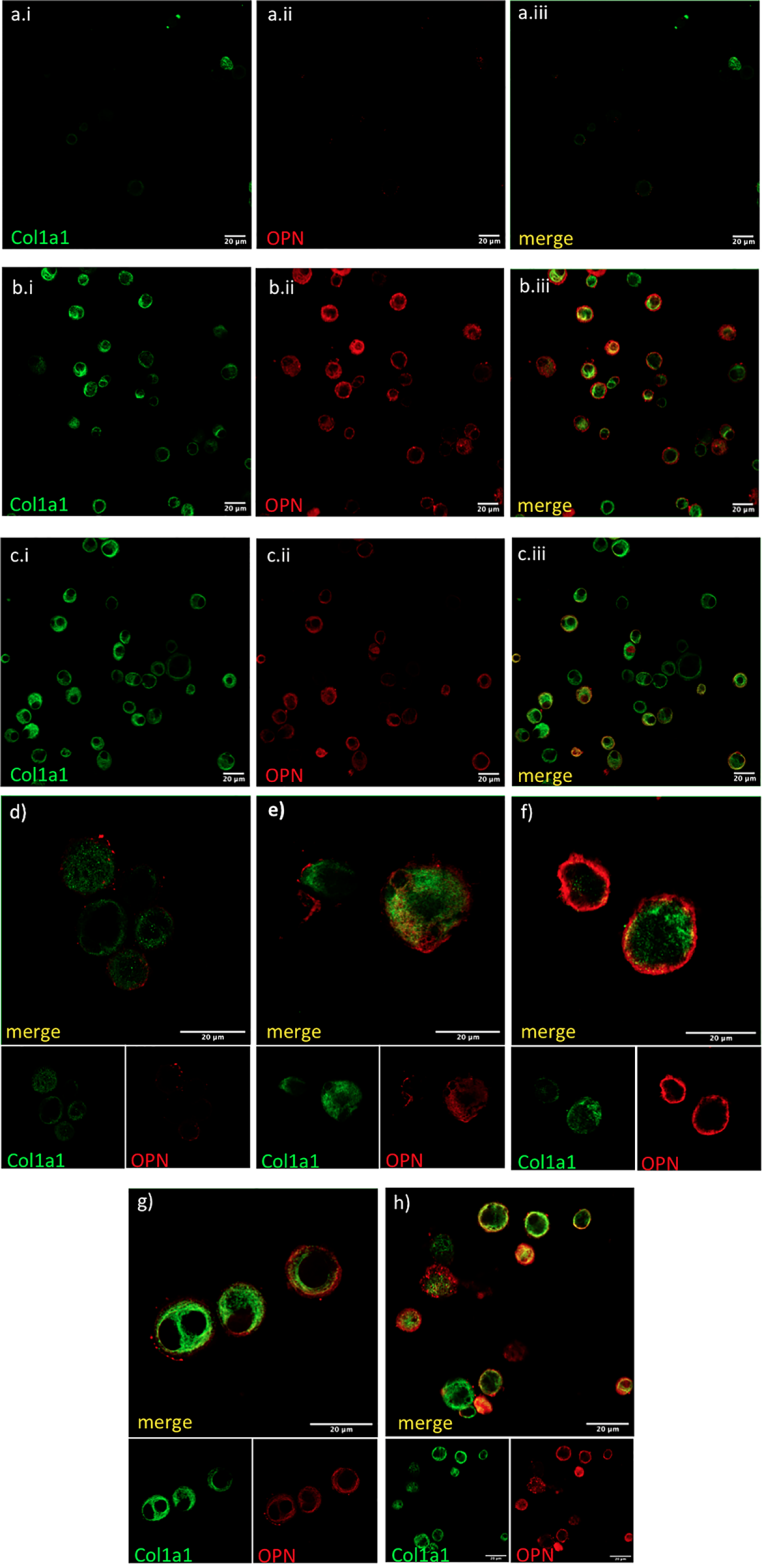
COL1A1 and OPN are coexpressed in AMs from fibrotic ILDs. Fresh BAL cultures were stained with rabbit anti-human-COL1A1and anti-human OPN in healthy **(A, D)**, IPF **(B, E, F)**, and RA-ILD **(C, G, H)**. Panels **(A.i, B.i, C.i)** show COL1A1 stained cells, panels **(A.ii, B.ii, C.ii)** show OPN stained cells and panels **(A.iii, B.iii,C.iii)** are the corresponding merged images. Panels **(D–H)** show merged COL1A1 and OPN images with corresponding single stainings as smaller insets below.

Finally, we tested if AMs co-expressed OPN and COL1A1. COL1A1 mRNA levels were moderately associated with higher SPP1 mRNA in f-ILDs other than IPF (Pearson’s R=0.36, p=0.006). In IPF, SPP1 levels although elevated, did not correlate with COL1A1. However, co-expression of OPN and COL1A1 could be observed in AMs in IPF and N-IPF BAL cultures as shown in **Figure 5**.

### Elevated COL1A1 mRNA in BAL Cells Is Associated With Worse Survival and ILD Progression

**Figure 6:**
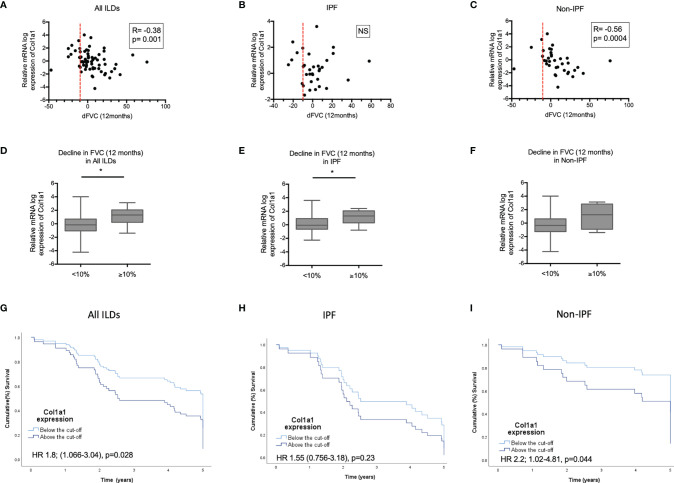
COL1A1 expression in relation to progression and mortality. Spearman correlation coefficient between relative mRNA expression of COL1A1 and FVC decline in 12 months in: **(A)** all ILDs combined (IPF and other ILDs), **(B)** IPF and **(C)** ILDs other than IPF. Comparison of COL1A1 relative mRNA expression in progressors (≥10% FVC decline at 12 months) and non progressors (<10% FVC decline at 12 months in **(D)** all ILDs, **(E)** IPF and **(F)** ILDs other than IPF unpaired T-test or Mann-Whitney tests. Kaplan Mayer Survival curves of patients with high (above the ROC curve-defined cut-off) and low expression of COL1A1 mRNA expression in: **(G)** all ILDs combined, **(H)** IPF and **(I)** ILDs other than IPF. (*p < 0.05). HR, Hazard ratio).

We subsequently tested possible associations of increased COL1A1 and SPP1 expression with disease severity in IPF and non-IPF patients. In the subgroup of patients with available serial PFTs (n=66), higher COL1A1 relative mRNA expression was associated with 12 month-FVC trends in the whole ILD population (R=-0.38, p=0.001, Pearson correlation). Subgroup analysis revealed that FVC decline was largely associated with higher COL1A1 mRNA in non-IPF patients (n=36) (R=-0.56, p=0.004, Spearman correlation), but not in IPF (n=33) (R=-0.084, p=NS, Spearman correlation) (**Figures 6** **A–C**).When ILD patients were dichotomized in two categories, stable and progressive ILD, as defined by FVC decline of 10% or more, progressive ILD patients had higher mean COL1A1 mRNA levels compared to stable ILDs (**Figures 6D–F**).

COL1A1 as a continuous variable was linked to higher mortality risk in ILDs (HR 1.3 CI 1.1-1.6, p=0.007), independently of age or disease severity at baseline, CPI (marker of severity) and UIP (independent predictor of worse survival) (HR 1.3; CI 1-1.7, p=0.02). Subgroup analysis of the IPF and N-IPF groups showed that COL1A1 was mostly associated with mortality in the non-IPF cohort with a HR ratio of 1.4 (CI: 1.1-1.8, p=0.01). After adjustment for age, gender, CPI and UIP pattern, COL1A1 remained independently associated with worse survival (HR: 1.6; CI 1.1-2.2 p=0.008) in Non-IPF patients. This was not observed in IPF (HR 1; CI 0.7-1.6, p=NS).

ROC curve-defined cut-off showed that a relative mRNA expression of log 0.5382 was associated with 1.8 times worse risk of mortality in all ILDs (HR 1.8; CI 1.06-3, p=0.03). This remained significant after adjusting for age (HR 1.8; CI 1.2-3.1, p=0.02) or CPI (HR 1.9; 1.1-3.3, p=0.02), but not UIP (HR 1.5 CI 0.8-2.7, p=0.6). Subgroup analysis revealed that the COL1A1 expression above this cut-off was associated with 2.2 times increased risk of death in the non-IPF ILD cohort (HR 2.2; CI 1-4.8, p=0.04) but not in IPF (HR 1.55; CI 0.7-3.2, p=NS). In the Non-IPF group this significant association was independent of age, CPI and UIP (HR 3.8; CI 1.4-10.2 p=0.01). Kaplan Meier curves for the patients with collagen expression above and below the cut-off are shown in **Figures 6** **G–I**.

SPP1 mRNA expression by airway macrophages in unadjusted analysis was not associated with survival in either group and after adjustment for age, CPI and UIP pattern, SPP1 associate with better outcomes in IPF (HR 0.6; CI 0.4-0.97 p=0.04) but not in the Non-IPF group (HR 0.9, CI 0.7-1.2, p=NS).

## Discussion

In this study, we show that alveolar macrophages express collagen 1a1 and this is associated with worse outcomes in ILDs. We examined a cohort of 115 ILD patients and found that the expression of COL1A1 increased in IPF and other progressive ILDs. A UIP pattern of fibrosis was characterised by higher COL1A1 expression in all ILDs and the higher expression was largely associated with worse survival in Non-IPF ILDs. COL1A1 and OPN were abundantly co-expressed in airway macrophages from all ILDs and were significantly higher relative to healthy individuals.

For years, IPF has been considered a distinct disease, of unknown cause, characterised by a UIP pattern of fibrosis and progressive fibrosis leading to dismal prognosis (41). New evidence suggests that some Non-IPF ILD patients may exhibit similar progression despite therapy and it is well known that Non-IPF ILDs can present with a UIP pattern of fibrosis. While the lumping of ILDs is controversial, it has been supported by recent evidence (3). Treatment with Nintedanib, a novel antifibrotic that changed the landscape in IPF treatment (42, 43), has similar efficacy in other PF-ILDs (44, 45), as well as in SSc-ILD (46). Similarly, Pirfenidone, another antifibrotic used in IPF (47), reduces FVC decline in unclassifiable ILDs (48). Based on the commonalities in clinical behaviour of IPF and PF-ILDs with the progressive fibrotic phenotype, it is speculated that common underlying pathogenetic pathways exist and drive fibrosis progression, despite variability of the initial trigger (6, 7).

Biomarkers that could predict progression in fibrotic ILD patients include UIP pattern on CT across different entities such as CTD-ILD (8, 49), hypersensitivity pneumonitis (9) and IPAF (50), severity of the disease at baseline such as DLCO, and/or CPI (51) and genetic or molecular biomarkers including MUC5b polymorphisms (52). Here we show that fibrotic ILD patients with a more progressive phenotype and in particular RA-ILD and NSIP patients, had higher COL1A1 expression than healthy individuals, much similar to IPF. Of particular interest, we showed that within the Non-IPF-ILD cohort higher COL1A1 expression in the BAL at the time of diagnosis was associated with more than two-fold risk of mortality, independently of a UIP pattern, disease severity and age. Similarly, we showed that patients with a UIP pattern of fibrosis, a classic imaging marker of progressive disease, have higher COL1A1 expression. BAL is a largely safe, minimally invasive procedure, used in most ILD centers worldwide for the diagnosis of ILDs (33). The discovery of this novel association between COL1A1 expression and survival could have implications in the management of ILDs, as BAL and COL1A1 measurement could act as a biomarker of progression at the initial stages of the ILD diagnosis. This would be a useful personalised medicine tool to stratify early, patients at risk of progression that would benefit from access to antifibrotic therapy before the detrimental progression occurs.

Although the expression of collagens by macrophages may be considered controversial it has been previously demonstrated in animal models. The COL1A1 expression atlas of fibrotic mouse lungs demonstrated that cells with hematopoietic marker CTPRC (encoding for CD45 antigen), CCR2 and CD68 macrophage markers expressed COL1A1 (53). Additionally, our re-analysis of alveolar macrophages isolated from mice fourteen days following bleomycin instillation (40), showed that COL1A1 was significantly upregulated relative to controls. We previously observed that mRNA levels of COL1A1 were detectable in whole BAL cell mRNA and were elevated in IPF patients relative to controls (24, 25). We also performed meta analyses of previously published BAL transcriptomic data from IPF patients (39) showing upregulation of COL1A1 in IPF BAL cells. In that study COL1A1 expression was associated with increased mortality, similarly to our study.

In order to exclude that this higher COL1A1 expression in the BAL might represent the presence of fibroblasts, we showed that COL1A1 was intracellularly expressed in airway macrophages with immunofluorescence and confocal microscopy. Cells with macrophage morphology isolated from BAL and positively stained for CD45 or CD163, were shown to contain high levels of COL1A1. CD163 positive macrophages accumulate in the tissue of fibrotic patients as previously shown (54) and in the BAL of IPF patients (24). According to previous reports that aimed at the identification of markers that would discriminate macrophages and fibrocytes from fibroblasts (21) CD45 was clearly not expressed on fibroblasts. In the same study, similar levels of COL1A1, CD45 and CD163 could be detected on macrophages and fibrocytes. Further characterisation is therefore needed in order to clarify the relative abundance of COL1A1, CD45 and CD163 positive macrophages and fibrocytes in the BAL of ILD patients.

An important finding of our study was that osteopontin and COL1A1 expression were both elevated in airway macrophages. Independent studies of lung macrophages using Single-cell RNAseq analyses have demonstrated the striking upregulation of SPP1 positive macrophages in IPF. Although the molecular mechanism of the induction of collagens by OPN is not thoroughly characterised, recent reports suggest that OPN acts through integrin α_v_β_3_ engagement and activation of the phosphoinositide 3-kinase/phosphorylated Akt/nuclear factor kappa B (PI3K/pAkt/NFκB)–signalling pathway at least in hepatic stellate cells (Urtasun et al. Hepatology 2012). Interestingly SPP1 levels correlated with COL1A1 expression in the Non-IPF ILDs but not within the IPF group suggesting a possible uncoupling of collagen expression from OPN in the IPF macrophages. Additionally, we observed that SPP1 mRNA was elevated in the BAL of most ILDs tested, irrespective of disease severity and CT patterns. Intriguingly, increased levels of SPP1 were associated with more favorable outcomes in IPF, in contrast to the rest of the ILDs. Circulating OPN was recently associated with immune complexes-driven profibrotic macrophage activity in SSc-ILD (55) and it could be speculated that OPN plays a more significant role in autoimmune-driven fibrosis as compared to IPF. Furthermore, recent studies with models of tissue repair and scar formation following heart injury showed that osteopontin expressing recruited macrophage populations play both a pro-fibrotic and pro-resolving role (56).

Our work also infers to a less characterised role of airway macrophages in pulmonary fibrosis, as it demonstrates a link between the macrophage derived expression of COL1A1 and fibrotic disease pathogenesis. There are few reports describing a direct role of macrophages in ECM production during fibrosis. A recent mouse model of fibrotic scar formation in the heart shows that recruited monocyte-derived macrophages to the injured site directly contribute to collagen-I fibril formation, suggesting, that collagen production is a component of the pro-fibrotic monocyte/macrophage response (22). Additionally, tumour associated macrophages of monocyte origin were shown to upregulate COL1A1 directly, affecting collagenous matrix organisation in the tumour microenvironment (57). In IPF, monocyte derived macrophages are also believed to play a detrimental role in the disease although the exact mechanism is not understood.

The main limitation of our study is the non-prospective validation of COL1A1 as a biomarker of ILD progression. This was mainly due to the small number of patients with non-IPF ILD and the small number of control patients. Additionally, further work is required for the precise elucidation of the macrophage populations that express COL1A1 as well as, their function. In conclusion our findings suggest that COL1A1 expression by macrophages may be a novel pathogenetic pathway in fibrotic ILDs that is related to worse outcomes. Our observations are exciting as COL1A1 expression could be further evaluated as a biomarker of Progressive ILD and raises hope for the early identification of patients that will develop a Progressive Fibrotic Phenotype.

## Data Availability Statement

The original contributions presented in the study are included in the article/**Supplementary Material**. Further inquiries can be directed to the corresponding author.

## Ethics Statement

The studies involving human participants were reviewed and approved by Ethics Committee of the University General Hospital of Heraklion and the Ethics Committee of Royal Brompton Hospital, London, UK.

## Author Contributions

ET and AT designed study, performed experiments, analyses and wrote the manuscript. EV, GM, and MK collected patient information and critically read the manuscript, SM performed experiments. DF performed meta-analyses from whole transcriptome data. AG performed mouse experiments. VA designed mouse experiments and critically read the manuscript. ER and NT recruited patients and critically read the manuscript. AW and KA designed study, recruited patients, and wrote manuscript. All authors contributed to the article and approved the submitted version.

## Funding

This work was supported by a Hellenic Thoracic Society (HTS) research award 2018 to ET and KA.

## Conflict of Interest

The authors declare that the research was conducted in the absence of any commercial or financial relationships that could be construed as a potential conflict of interest.

## Publisher’s Note

All claims expressed in this article are solely those of the authors and do not necessarily represent those of their affiliated organizations, or those of the publisher, the editors and the reviewers. Any product that may be evaluated in this article, or claim that may be made by its manufacturer, is not guaranteed or endorsed by the publisher.

## Abbreviations

AMs, Airway macrophages; COL1A1, Collagen 1a1; CTD-ILD, Connective tissue associated ILD; GAPDH, glyceraldehyde-3-phosphate dehydrogenase; HP, Hypersensitivity Pneumonitis; ILD, Interstitial Lung Diseases; IPF, Idiopathic Pulmonary Fibrosis; NSIP, Non-Specific Pneumonia; OPN, osteopontin; OP, organising pneumonia; RA-ILD, Rheumatoid Arthritis ILD; SSc-ILD, Scleroderma ILD; SLE, Systemic Lupus erythematosus; SPP1, secreted phosphoprotein 1; UIP, Usual Interstitial Pattern.
