# Supplementary material for: Collagen 1a1 Expression by Airway Macrophages Increases In Fibrotic ILDs and Is Associated With FVC Decline and Increased Mortality
Source: Front Immunol. 2021 Nov 17;12:645548. doi: 10.3389/fimmu.2021.645548 (PMC8635798; doi:10.3389/fimmu.2021.645548)
Supplement: Supplementary file 1 [file DataSheet_1.pdf]

Supplementary Material

1) Supplementary methods

Re-analysis of microarray and RNA-seq data Microarray and RNA-seq raw data were retrieved from GSE70866 (1) and GSE98468 (2), respectively. Background correction and quantile normalization of the Freiburg cohort microarray data were performed using *normexp* method and quantile normalization as implemented into limma Bioconductor package (Ritchie et al., 2015). Outlier samples were removed based on principal component analysis (PCA) plot created with arrayQualityMetrics Bioconductor package (Kauffmann et al., 2009). Probe intensity values were summarized at the gene level using a weighted average (weights sum up to the unit), post to control and multi-gene matching probes removal. Differentially expressed genes were recovered using the limma moderated t-test statistic with a fold change threshold of at least 1.2 (or below 0.8) and an FDR corrected p-value of less than 0.05. Raw RNA-seq data were fetched and pre-processed using the SRA toolkit, prior to mapping to GRCm38 mouse genome assembly using a two-tier alignment pipeline based on HISAT2 (v.2.1.0) (Kim et al., 2019) and Bowtie2 (v.2.3.5.1) (Langmead & Salzberg, 2012) aligners. Downstream analysis was performed using metaseqR2 Bioconductor package (Fanidis & Moulos, 2020). More specifically, aligned reads were quantified and then normalized using EDASeq normalization. After filtering using default parameters, differential expression analysis statistics were calculated using PANDORA p-value combination algorithm based on the results of DESeq, DESeq2, limma, edgeR and ABSSeq statistical analysis methods. The same differential expression thresholds as in microarray data were applied. For both analyses, proper outlier removal was validated with samples hierarchical clustering using the 1000 top differentially expressed genes. Analyses were performed in R version 3.6.1.

Fanidis, D., & Moulos, P. (2020). Integrative, normalization-insusceptible statistical analysis of RNA-Seq data, with improved differential expression and unbiased downstream functional analysis. *Briefings in Bioinformatics*. https://doi.org/10.1093/bib/bbaa156

Kauffmann, A., Gentleman, R., & Huber, W. (2009). arrayQualityMetrics - A bioconductor package for quality assessment of microarray data. *Bioinformatics*, *25*(3), 415–416. https://doi.org/10.1093/bioinformatics/btn647

Kim, D., Paggi, J. M., Park, C., Bennett, C., & Salzberg, S. L. (2019). Graph-based genome alignment and genotyping with HISAT2 and HISAT-genotype. *Nature Biotechnology*, *37*(8), 907–915. https://doi.org/10.1038/s41587-019-0201-4

Langmead, B., & Salzberg, S. L. (2012). Fast gapped-read alignment with Bowtie 2. *Nature Methods*, *9*(4), 357–359. https://doi.org/10.1038/nmeth.1923

Ritchie, M. E., Phipson, B., Wu, D., Hu, Y., Law, C. W., Shi, W., & Smyth, G. K. (2015). limma powers differential expression analyses for RNA-sequencing and microarray studies. *Nucleic Acids Research*, *43*(7), e47. https://doi.org/10.1093/nar/gkv007

2) Supplementary Figures and Tables

Supplementary Figure 1.

a) b) c)

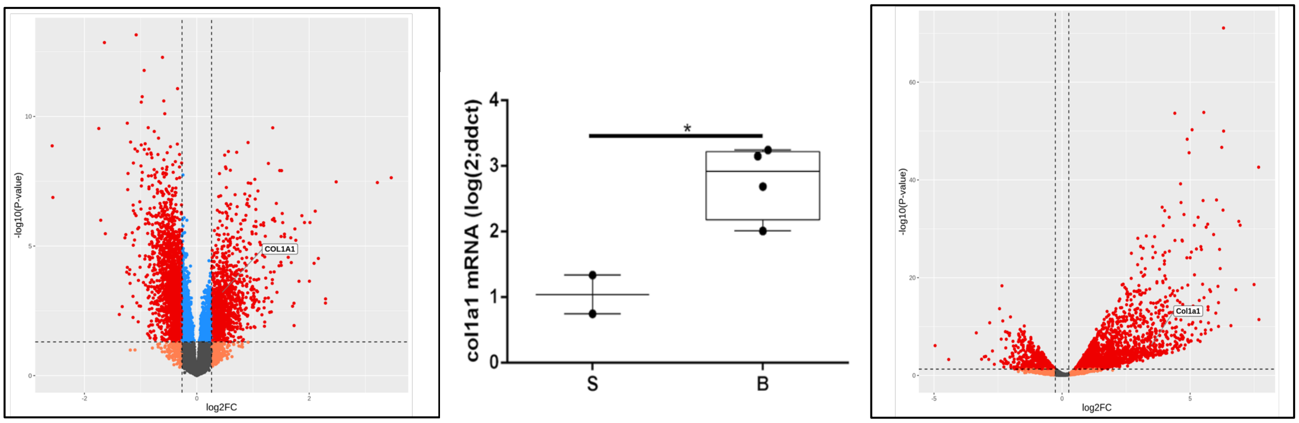

Meta-analysis of human BAL cells and mouse alveolar macrophages expression data and col1a1 mRNA levels in cells from mouse BAL.

a) Volcano plot of human BAL microarray transcriptomic data (GSE70866) demonstrates that COL1A1 is significantly overexpressed in IPF patients relative to healthy individuals (0.41 log2FC; 0.0007 pvalue; 0.008 FDR corrected pvalue). b) Relative col1a1 mRNA expression in cells from mouse BAL following instillation with bleomycin (B) or saline (S). c) Volcano plot of mouse alveolar macrophages RNA seq data (GSE98468) depicts Col1a1 up-regulation after 14 days of bleomycin instillation (3.41 log2FC; 1.38e-06 p value; 4.4e-05 FDR corrected p value). (*p value<0,05).

Supplementary Figure 2.

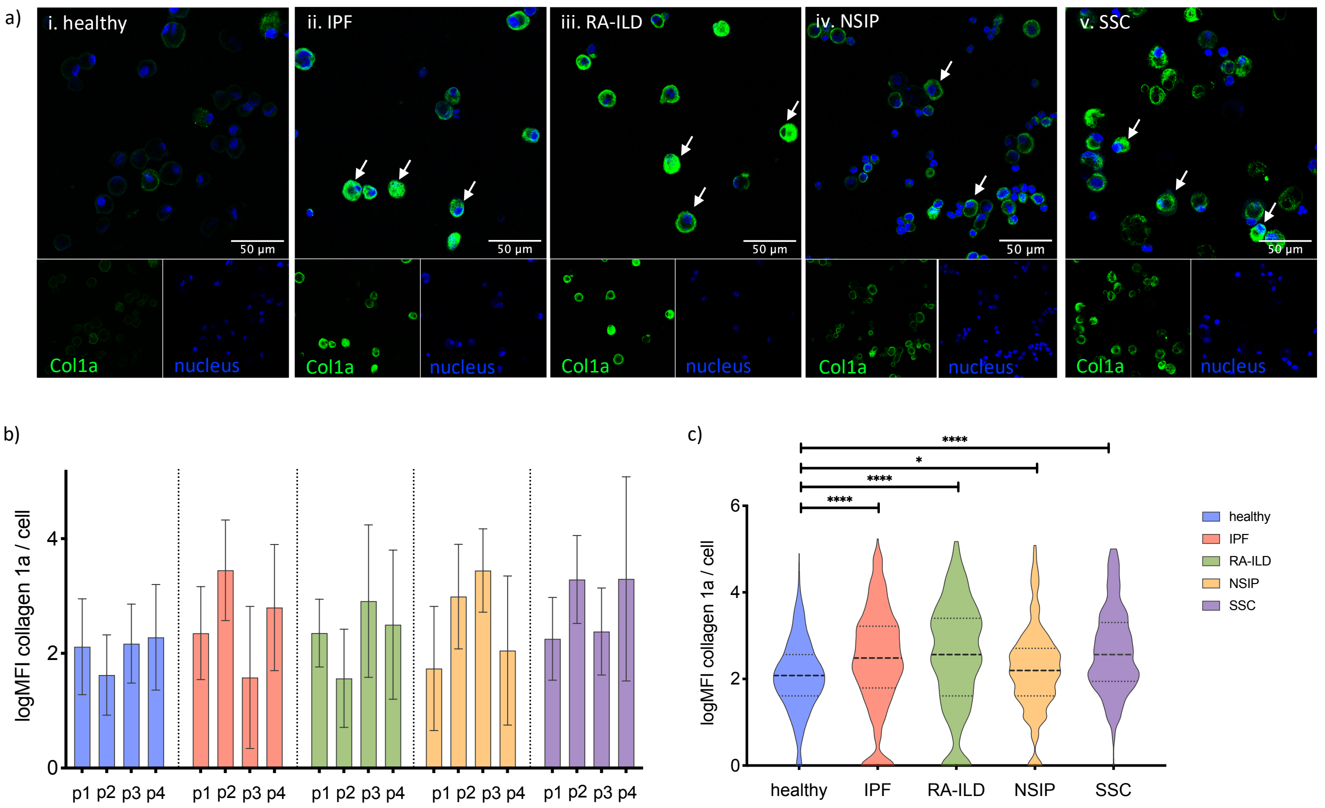

Expression levels of COL1A1 in AMs the ILD diagnostic subgroups relative to heathy. a) Images of BAL cytospins stained with anti-human Col1a1 and ToPro-633 nuclear stain in (i) healthy, (ii) IPF, (iii) RA-ILD, (iv) NSIP, and (v) SSc-ILD. Arrows indicate prominent collagen1a expression in BAL cells. b) mean logCol1a1 expression/cell per patient tested. c) Violin plots of mean logCol1a1 expression/cell per disease group. Kruskal-Wallis test relative to healthy $p<0.0001$, ****= $p<0.0001$, * =$P< 0.05$ (healthy vs NSIP $p=0.024$)

Supplementary Figure 3.

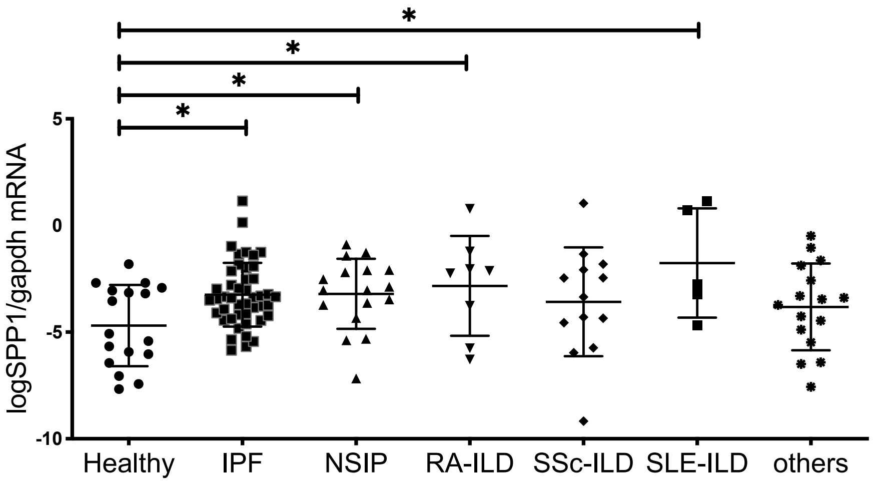

SPP1 expression in whole BAL in the ILD diagnostic subgroups relative to heathy. Individual comparisons relative to healthy, IPF, p=0.03, NSIP, p=0.03, RA-ILD, p=0.04, SLE-ILD, p=0.022. (healthy n=17, NSIP n=19, RA-ILD n=8, SSc-ILD n=12, SLE-ILD n=6 and others n= 16)

Supplementary Figure 4.

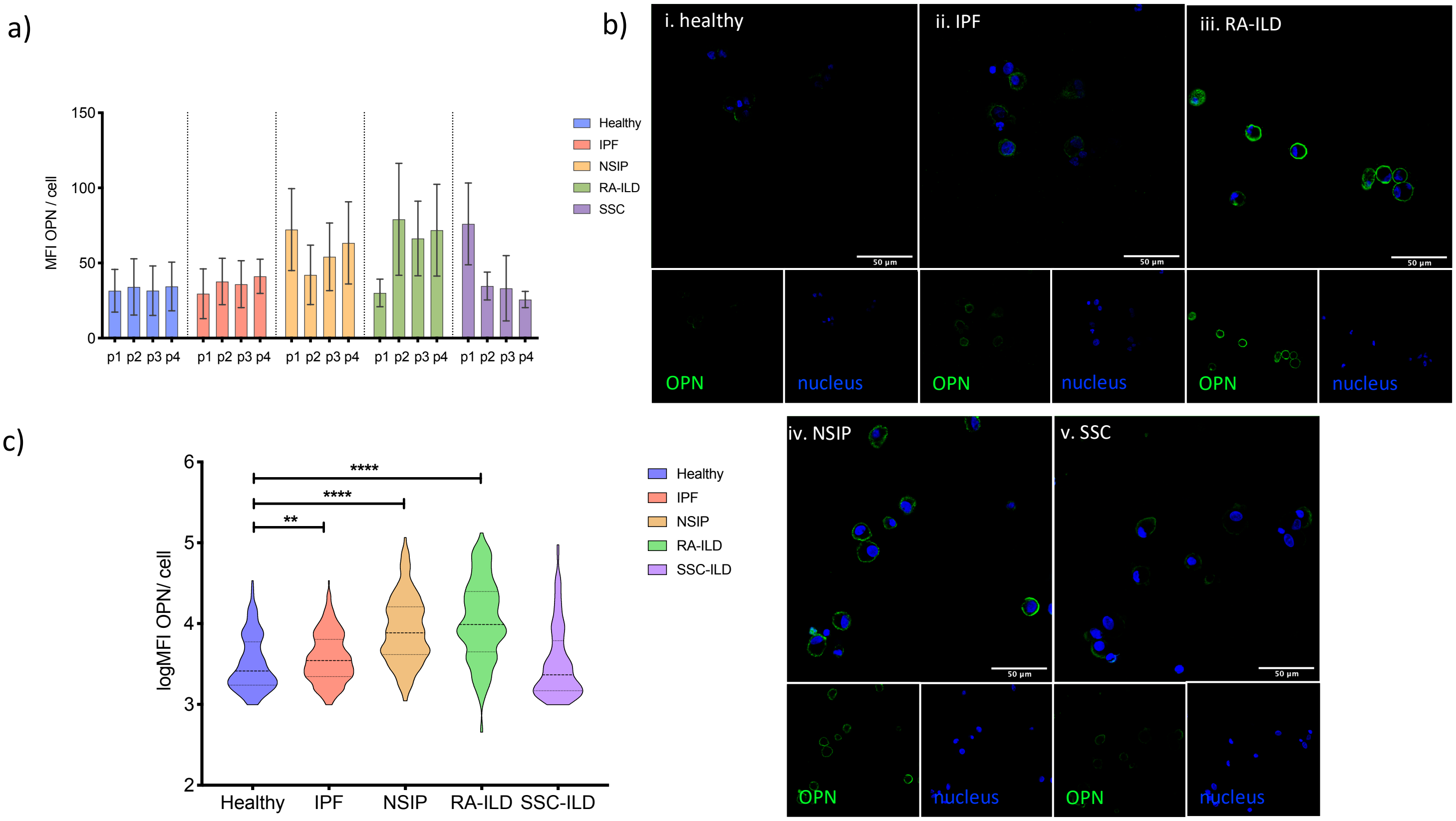

OPN expression in AMs. a) Mean OPN expression per/cell in each patient. b) Representative images of BAL cytospins stained with anti-human OPN and ToPro-633 nuclear stain in (i) healthy, (ii) IPF, (iii) RA-ILD, (iv) NSIP, and (v) SSc-ILD. c) Violin plots of log mean fluorescence intensity of OPN /cell per disease group. (Kruskal-Wallis test relative to healthy ** =p< 0.01, ****= p< 0.0001)

Supplementary Figure 5.

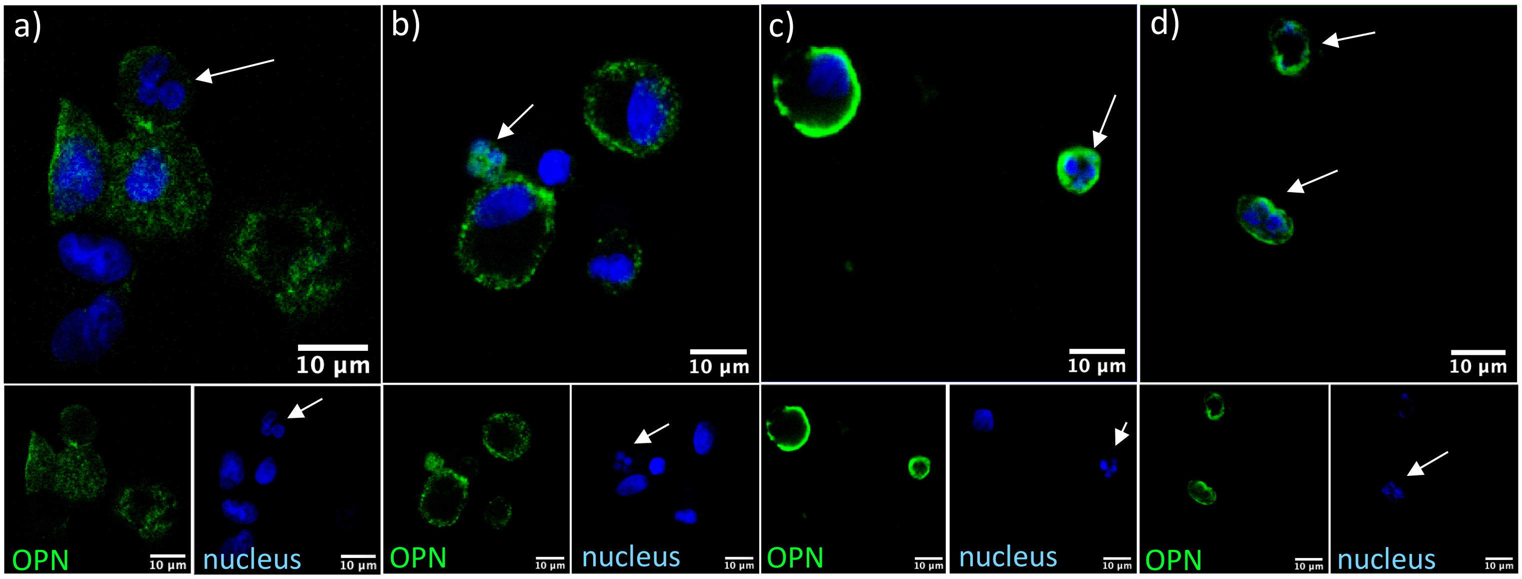

OPN expression in neutrophils.

Representative images of BAL cytospins stained with anti-human OPN and ToPro-633 nuclear stain from (a) IPF, (b) NSIP, (c) RA-ILD and (d) SSC patients. Arrows indicate neutrophils, identification based on nuclear morphology.

Supplementary Table 1

	Control Group	IPF	NSIP	RA-ILD	Scleroderma-ILD	SLE-ILD	Other ILDs*
N:	19	53	19	8	13	5	17
Age	54.1 ±13.5	68.1 ±10	60±11	66±6	54±16.6	51.7±15.6	62.8±15.5
gender (female/ male)	6/13	8/45	13/6	5/3	7/6	4/1	10/5
Macrophages	87.3±9	79.1±15.3	76±13	77±14	78.6±14	79.4±9	81±13
Lymphocytes	9.9±7.6	11±15.2	13±8	10±6	10.9±11.7	10.5±7.2	10±10.9
Neutrophils	4.2±1.6	7.3±6.3	5.8±4	9±8.6	6.5±8	7.3±7	5.7±5.3
Eosinophils	0.4±0.5	2±3.3	3.6±4	2.3±3.1	2.2±2.4	1±0.6	1.9±2
FVC		78.5±19.7					
DLco		52.3±18.5	45±14	57.9±14.8	45±13	48.4±10.3	58.6±19
CPI		44±15	45±15	37.6±11	46.5±14	42.7±15	38.7±16
